# Nutritional Factors Associated with Anaemia in Pregnant Women in Northern Nigeria

**Published:** 2007-03

**Authors:** Dorothy J. VanderJagt, Hugh S. Brock, George S. Melah, Aliyu U. El-Nafaty, Michael J. Crossey, Robert H. Glew

**Affiliations:** ^1^ Department of Biochemistry and Molecular Biology, University of New Mexico School of Medicine, Albuquerque, NM, USA; ^2^ Department of Obstetrics and Gynaecology, Federal Medical Centre, Gombe, Nigeria; ^3^ TriCor Reference Laboratory, Albuquerque, NM

**Keywords:** Anaemia, Iron-deficiency, Pregnancy, Homocysteine, Folate, Vitamin B12, Cross-sectional studies, Nigeria

## Abstract

This study was conducted to assess the relative contribution of iron, folate, and B12 deficiency to anaemia in pregnant women in sub-Saharan Africa. In total, 146 pregnant women, who attended two antenatal clinics in Gombe, Nigeria, were recruited into the study. The majority (54%) of the women were in the third trimester. Blood samples were obtained for determination of haematocrit and for measurement of serum iron, total iron-binding capacity, ferritin, folate, vitamin B12, and homocysteine. Malaria was present in 15 (9.4%) women. Based on a haemoglobin value of <105 g/L, 44 (30%) women were classified as anaemic. The major contributing factor to anaemia was iron deficiency based on the serum concentration of ferritin (<10 ng/mL). The mean homocysteine concentration for all subjects was 14.1 μmol/L, and homocysteine concentrations were inversely correlated with concentrations of folate and vitamin B12. The serum homocysteine increased markedly at serum vitamin B12 levels below 250 pmol/L. The most common cause of anaemia in the pregnant women in northern Nigeria was iron deficiency, and the elevated concentrations of homocysteine were most likely due to both their marginal folate and vitamin B12 status.

## INTRODUCTION

Anaemia in pregnancy is a global problem and is associated with increased maternal morbidity and mortality (1). Anaemia in pregnancy is more common in developing countries than in developed countries. In the USA, less than 30% of pregnant women develop anaemia, whereas the prevalence rates in Africa, Asia, and Latin America range from 35% to 75% (2, 3). Maternal deaths from anaemia range from 34 per 100,000 livebirths in Nigeria to as high as 194 per 100,000 livebirths in Pakistan (1, 4).

In 1987, the United Nations Children's Fund and re-presentatives from 45 countries launched the Safe Motherhood Initiative (SMI) for reducing maternal mortality worldwide by 50% by 2000 (5). One of the major goals of the Initiative was to eradicate anaemia during pregnancy. Results of recent studies of maternal morbidity and mortality in sub-Saharan Africa showed that anaemia continues to be a major contributing factor to maternal mortality in this part of the world (6–8).

In developing countries, the cause of anaemia during pregnancy is multi-factorial and includes nutritional deficiencies of iron, folate, and vitamin B12 and also parasitic diseases, such as malaria and hookworm. The relative contribution of each of these factors to anaemia during pregnancy varies greatly by geographical location, season, and dietary practice. In sub-Saharan Africa, iron and folate deficiencies are the most common causes of anaemia in pregnant women (9–11). Vitamin B12 deficiency may be an unrecognized contributor to anaemia in this region of the world due to reliance of the population on grains as dietary staples and low consumption of foods of animal origin which are the primary source of dietary vitamin B12. We have documented that approximately 10% of adolescent girls in Maidudguri, Nigeria, had serum vitamin B12 levels, indicative of a deficiency of this vitamin (12).

The purposes of this cross-sectional study were to determine the incidence of anaemia among pregnant women attending typical antenatal clinics in northern Nigeria and to inquire about the contribution of iron, folate and vitamin B12 deficiencies to anaemia in these women.

## MATERIALS AND METHODS

### Subjects

A cross-sectional study of pregnant women who presented at two antenatal clinics (the Federal Medical Centre and the Specialist Hospital) in Gombe, Nigeria, was conducted during June-August 2003, which corresponds to the rainy season. These clinics are typical of antenatal clinics elsewhere in northern Nigeria. The women were inhabitants of the town of Gombe and villages that lie within a 30-mile radius of the town. The study was limited to healthy normotensive pregnant women with no history of hypertension, proteinuria, or other complications of pregnancy. Women with pre-eclampsia (systolic blood pressure ≥140 mm Hg or diastolic blood pressure ≥90 mm Hg) and a positive urine protein (measured by dip-stick) were excluded from the study.

A single blood sample for biochemical analyses was obtained from each subject who visited the antenatal clinic during June-August 2003. A haematocrit was obtained for each subject at the time of her visit to the antenatal clinic. Informed consents were obtained from all the subjects after describing the study, and its requirements were given to them in English or their native language (*Hausa* or *Fulfulde*) by a translator. At the time the blood sample was drawn, information regarding gravidum, parity, education, and use of supplements by the subjects was recorded. Gestational age was estimated by both using date of the last menstrual period and measuring fundal height. The presence of malarial infection was determined by microscopy using thick smears. Since biochemical analyses were not completed until several months after the blood samples were drawn, it was not possible to judicate administration of supplements on the basis of specific nutrient deficiencies of iron, folate, or vitamin B12. However, it is a standard procedure at these two antenatal clinics to provide iron and folate supplements to any pregnant woman whose haematocrit is indicative of anaemia.

The Human Research Review Committee of the University of New Mexico School of Medicine and the Ethics Review Committee of the Federal Medical Centre, Gombe, approved the study.

### Anthropometric measurements

Age in years was self-reported. Weight was measured using a battery-operated scale accurate to 0.5 kg, and height was measured to within 0.25 cm using a portable stadiometer. Mid-arm circumference was determined using a tape (Creative Health Products, Plymouth, MI, USA). Triceps skin-fold thickness was measured with a body caliper (Caliper Company, Inc, Carson City, NV, USA).

### Biochemical analyses

Blood was obtained by venipuncture and collected into trace metal-free vacutainer tubes containing EDTA (Becton Dickinson, New Jersey, USA). After determining the haematocrit (packed cell volume), the plasma fraction was obtained by centrifugation, aliquoted into cryovials, and stored at -80 °C. Samples were transported to Albuquerque, NM in the frozen state for analysis of iron, total iron-binding capacity, ferritin, folate, vitamin B12, and homocysteine.

Serum iron and unsaturated iron-binding capacity were measured using the Ferrozine colorimetric assay (Boehringer-Mannheim Diagnostics, Hitachi model 717, Indianapolis, IN; detection limit 5 μg/dL and 10 μg/dL respectively). Total iron-binding capacity was calculated as the sum of serum concentration of iron and unsaturated iron-binding capacity. The percentage of iron saturation was calculated from the serum concentration of iron and total iron-binding capacity. Plasma folate and vitamin B12 were determined using a chemiluminescent immunological assay (Diagnostic Products Corporation, Immulite 2000, Los Angeles, CA, USA; detection limit 0.3 ng/mL and 50 pg/mL respectively). Plasma homocysteine was measured using a flouresence polarization immunological assay (Abbott Diagnostics, AxSYM System®, Abbott Park, IL, USA; detection limit 0.8 μmol/L). Serum ferritin was measured using a che-miluminescence assay (Beckman Diagnostics, Access B, Fullerton, CA, USA; detection limit 0.2 ng/mL).

### Statistical analysis

Unless otherwise indicated, results are expressed as the mean±1 standard deviation. For those variables that were not normally distributed, results are given as the median followed by the range indicated in parentheses. Parameters with a non-normal distribution were trans-formed prior to statistical analyses. Descriptive statistics, group comparisons, and correlation analyses were made using the Number Cruncher statistical software (NCSS, Kaysville, UT, USA). A p value of 0.05 was considered to be statistically significant.

## RESULTS

### Study population

In total, 146 pregnant women were enrolled in the study. Their anthropometric characteristics are summarized in Table 1. Their mean age was 26.7 (range 15–45) years, their mean height was 160.2 cm, and their mean weight was 62.8 kg. The majority (n=79, 54%) were in the third trimester of pregnancy with 5% (n=8) in the first trimester and 41% (n=59) in the second trimester. Gravidum ranged from 1 to 17 with a median of 5. Parity ranged from 0 to 11 with a median of 3 children. Forty-two percent of the subjects took iron supplements, 30% folate supplements, and 33% multi-vitamins. Fifteen (9.4%) women tested positive for malaria by microscopy of thick blood smears at the time of the study. Approximately, 25% (n=37) of the women took malaria prophylaxis. The majority (61%) reported having had no formal education.

**Table 1.** Anthropometric characteristics of pregnant women in Gombe, Nigeria

Parameter	Trimester 1(n=8)	Trimester 2 (n=59)	Trimester 3 (n=79)
Age (years)	23.0± 7.1*	26.7±6.4	27.5±6.0
Height (cm)	161± 5	159±68	160±6
Weight (kg)	62.1±14.7	61.5±12.7	63.9±11.6
MAC (cm)	25.3±4.3	26.2±3.9	26.2±3.4
TSF (mm)	15±7	18±8	20±6
Gestation (weeks)	8.2±3.6	21.4±3.5	32.5±3.1
Gravida	4 (1–10)†	5 (1–12)	5 (1–17)
Parity	0 (0–9)	3 (0–8)	4 (0–11)

*Mean±SD;

†Median (range)

MAC=Mid-arm circumference;

TSF=Triceps skin-fold thickness

### Biochemical analyses

Results of biochemical analysis are summarized in Table 2. Because of the haemodilution that naturally occurs during pregnancy, the results of biochemical analyses are grouped according to trimester. The mean concentration of haemoglobin for the 146 subjects was 110 g/L, with a range of 50 g/L to 180 g/L. Overall, 44 (32%) had a haemoglobin concentration below 105 g/L, the cut-off for anaemia established by the World Health Organization (4). The lowest mean concentration of haemoglobin (109 g/L) was seen in women in the third trimester. However, there was no statistically significant difference in haemoglobin levels by trimester. Although the total iron-binding capacity increased significantly between the second and the third trimester, there was no difference in serum concentration of iron, percentage of transferrin saturation, or ferritin concentration among trimesters.

**Table 2.** Biochemical characteristics of pregnant women in Gombe

Parameter	Trimester 1 (n=8)	Trimester 2 (n=59)	Trimester 3 (n=79)	Normal values in pregnancy
Haemoglobin (g/L)	116±18*	112±17	109±14	>105†
Haematocrit (%)	35±5.5	34±5.1	33±4.3	>31.5†
Iron (μg/dL)	101±31	104±52	99±44	47–160‡
TIBC (pg/mL)	421±98	427±83	466±82	348–591‡
Iron saturation (%)	26±11	26±14	22±13	10.2–34.0‡
Ferritin (ng/mL)	27.7 (7.8–94.6)*	22.3 (6.3–314)	16.4 (4.0–206)	>10‡,>30¶
Folate (nmol/L)	17.9±7.9	17.3±8.4	18.3±9.5	>7.7‡
Vitamin B12 (pmol/L)	240±59	228±76	218±66	148–616‡
Homocysteine (μmol/L)	14.2±5.1	12.9±3.5	14.0±4.9	4–12**

*Mean±standard deviation;

**Median (range);

TIBC=Total iron-binding capacity;

†World Health Organization;

‡Burtis CA, Ashwood ER. Tietz Fundamentals of clinical chemistry (13);

¶van den Broek *et al. Br J Haematol* 1998;103:817–24 (14)

Twenty seven (18%) women had a serum ferritin concentration of <10 ng/mL, a level which is considered indicative of iron deficiency in non-pregnant women. Sixteen (20%) women in their third trimester had ferritin levels below 10 ng/mL, and 10 (16%) subjects had ferritin levels below 10 ng/mL in their second trimester. In an investigation of parameters used for screening iron status in pregnant women, van den Broek and co-workers (14) determined that a ferritin value of 30 ng/mL or less was the best indicator of iron deficiency in pregnant women when using a stained marrow biopsy for validation. Applying a cut-off of 30 ng/mL for iron deficiency in the present study, 64% (n=94) of the subjects were classified as iron-deficient—33 (35%) in the second trimester and 57 (61%) in the third trimester.

The serum concentrations of folate and vitamin B12 of the pregnant women in each trimester are shown in Table 2. The mean concentration of folate for the 146 subjects was 17.9 nmol/L. Only 13 women had a serum concentration of folate below 7.7 nmol/L, a concentration that is at the lower end of normal for folate. There were no differences in the serum concentrations of folate among groups. The mean concentration of vitamin B12 for the subjects was 223 pmol/L, which is above the cut-off of 148 pmol/L for deficiency. However, 15 (9%) of the women had a vitamin B12 concentration below this cut-off. There were no significant differences in the mean concentrations of vitamin B12 based on trimester.

**Fig. 1.**
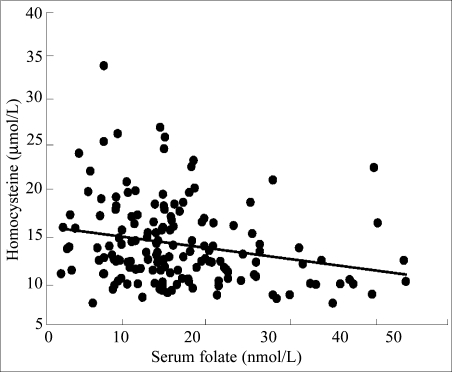
Relationship between serum concentrations of homocysteine and serum concentrations of folate in pregnant women in northern Nigeria (r=0.24, p=0.003)

**Fig. 2.**
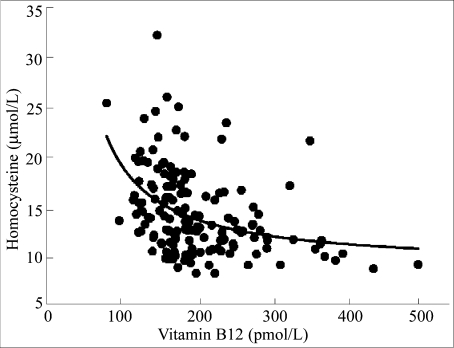
Relationship between serum concentrations of homocysteine and serum concentrations of vitamin B12 in pregnant women in northern Nigeria (r=0.41, p<0.001)

Homocysteine can be elevated in serum as the result of either folate or vitamin B12 deficiency. Eighty-eight (55%) women had a serum concentration of homocysteine above 12 μmol/L, and the homocysteine levels did not differ by trimester. Significant negative relationships with homocysteine were obtained for both folate (Fig. 1) and vitamin B12 (Fig. 2). The relationship between homocysteine and folate was best described by a linear equation (Fig. 1, r=0.24, p=0.003), whereas the relationship between homocysteine and vitamin B12 was best described by non-linear equation in which serum homocysteine concentrations increased markedly below 250 pmol/L (Fig. 2, r=0.41, p<0.001).

Subjects were next grouped according to haemoglobin level (<105 and ≥105 g/L), and the gestational age-adjusted means for the biochemical parameters are shown in Table 3. The only biochemical parameter besides haemoglobin concentration and haematocrit that differed between the two groups was serum iron. The anaemic subjects had a mean gestational age-adjusted ferritin concentration of 13.5 ng/mL compared to 22.3 ng/mL for the non-anaemic women.

**Table 3.** Biochemical values for women with haemoglobin values < or ≥105 g/L

Parameter (mean±SD)	Haemoglobin (g/L)	
	<105 (n=44)	≥105 (n=102)
Haemoglobin	93.9 (1.6)	117 (1.1)*
Haematocrit (%)	28.3 (0.49)	35.4 (0.32)*
Iron (μg/dL)	89 (7.0)	106 (4.6)**
TIBC (pg/mL)	453 (12.5)	446 (8.2)
Iron saturation (%)	22 (2.0)	25 (1.3)
Ferritin (ng/mL)	34.1 (5.9)	31.9 (3.9)
Folate (nmol/L)	15.9 (1.37)	18.7 (0.89)
Vitamin B12 (pmol/L)	209 (10.5)	230 (6.9)
Homocysteine (μmol/L)	14.1 (0.66)	13.3 (0.43)

*p<0.001;

**p=0.04

TIBC=Total iron-binding capacity

Twelve (24%) of the 44 women who were classified as anaemic based on a haemoglobin level below 105 g/L had a serum concentration of ferritin below 10 ng/mL, and 33 (75%) had a serum concentration of ferritin below 30 ng/mL (Table 4). Of the subjects with anaemia, 27% had low ferritin levels, 14% had a sub-normal folate concentration, and 9% had a vitamin B12 concentration of <148 pmol/L. These data suggest that iron deficiency was the major cause of anaemia among our study women. Of the non-anaemic subjects (n=102), 15% had a sub-normal ferritin level, 11% had low folate levels (<7.7 nmol/L), and 7% had a serum vitamin B12 level indicative of a deficiency of this vitamin. Although more of the anaemic subjects in our study had evidence of iron deficiency based on serum ferritin levels, we were interested in the relative contribution of other nutritional factors (i.e. folate, vitamin B12) which may have lead to anaemia in these pregnant women. Using a multiple regression model which included ferritin, folate, vitamin B12, and gestational age as the independent variables, we found that only gestational age significantly correlated with haemoglobin concentration.

**Table 4.** Number and percentages of pregnant women with biochemical values indicative of deficiency

Parameter	Haemoglobin <105 g/L (n=44)		Haemoglobin ≥105 g/L (n=102)	
	No.	%	No.	%
Ferritin (<10 ng/mL)	12	27	15	15
(<30 ng/mL)	33	75	61	42
Folate (<7.7 nmol/L)	6	14	7	7
Vitamin B12 (<148 pmol/L)	4	9	11	11
Malaria-positive	3	7	12	12

Twenty-two women with a haemoglobin level of <105 g/L took iron supplements, 13 took folate supplements, and 9 took both iron and folate supplements. Only three anaemic women tested positive for malaria (Table 4). Malaria was detected in 22 % of the non-anaemic women.

## DISCUSSION

In Gombe, Nigeria, and its environs, most prenatal care is provided collectively by the Federal Medical Centre, a tertiary-care centre, and the Specialist Hospital which serves patients from a broad range of socioeconomic levels. The goals of this study were to determine the prevalence of anaemia and to examine the contributions of iron, folate and vitamin B12 status to anaemia in pregnant women residing in this region. Our main finding was that 30% of the pregnant women were anaemic, using the WHO cut-off haemoglobin concentration of 105 g/L. The most common deficiency relating to anaemia in these women was iron.

This finding was not unexpected, since the diets of the population in the region are heavily reliant on grains, such as millet and sorghum, that contain large amounts of phytates which are known to interfere with the intestinal uptake of iron and other trace minerals, such as zinc and calcium. Requirements of iron during pregnancy are high, and it is difficult to meet the requirements through diet alone. Therefore, maintaining iron balance during pregnancy is dependent on maternal iron stores.

To better understand the aetiology of anaemia in our study population, we determined the proportion of anaemic and non-anaemic women who were deficient in iron (ferritin <30 ng/mL), folate (<7.7 nmol/L), or vitamin B12 (<148 pmol/L). The greatest distinction between the anaemic and the non-anaemic subjects was in the serum ferritin level: 27% of the anaemic women had serum ferritin concentrations of <10 ng/mL compared to 16.4% of the non-anaemic women. On the other hand, the incidence of vitamin B12 deficiency was similar in the anaemic (7.8%) and non-anaemic groups (10.9%), and the numbers of subjects with folate deficiency were comparable in both the groups (Table 4). Malaria was not common in women who were classified as anaemic; only 4 of the 44 anaemic women had malaria at the time of the study.

These results are similar to those reported for anaemic pregnant women in Malawi where van den Broek and Letsky (15) reported that 23% of anaemic pregnant women in the southern part of the country were deficient in iron. One-third of anaemic subjects in that study were also deficient in vitamin B12, while another one-third were deficient in folate. Malaria and hookworm infections were found in 8% and 6% of their subjects respectively.

The most common screening test for assessing an individual's vitamin B12 status is the serum concentration of vitamin B12. Serum vitamin B12 concentrations have been shown to lack sensitivity in detecting vitamin B12 deficiency. Assays for vitamin B12, such as competitive protein-binding immunoassay used in this study, detect all forms of vitamin B12 in serum, including physiologically-inactive analogues. On the other hand, excretion of increased amounts of methylmalonic acid in urine is an early indicator of vitamin B12 deficiency. In a study of elderly individuals in the USA, 10–40% of the subjects had elevated methylmalonic acid in their urine, while their serum concentration of vitamin B12 was in the low-normal to normal range (16). Although the vitamin B12 concentration in the sera of most women in this study was above 148 pmol/L, the lower end of the reference range, the serum concentrations we observed in this study, may not reflect the physio-logically-active amount of vitamin B12 in our subjects studied.

The second important finding of the study was the high percentage of pregnant women with an elevated serum concentration of homocysteine. The mean homocysteine level (14.1 μmol/L) of pregnant women in Gombe exceeded the upper limit of normal (i.e. 12.0 μmol/L) (16). Since both folate and vitamin B12 are involved in a single-carbon transfer reaction that converts homocysteine to methionine, a deficiency of either vitamin can cause the serum concentration of homocysteine to be elevated. An elevated homocysteine level during pregnancy is associated with several adverse outcomes, including increased habitual spontaneous abortions, placental abruption, and preeclampsia (17, 18). Several of our previous studies with adolescent girls and adults of both genders have shown that moderate hyperhomo-cysteinaemia was prevalent in Nigerian populations (12, 19, 20). We also reported that Nigerian women with pre-eclampsia had significantly higher serum concentrations of homocysteine than their healthy counterparts (10.1 vs 8.4 μmol/L), and their homocysteine concentrations were inversely correlated with high-density lipoprotein concentrations (21). In the present study, serum concentrations of homocysteine correlated inversely with both folate and vitamin B12 levels (Figs. 1 and 2). Of particular interest was the marked increase in the serum concentrations of homocysteine when the vitamin B12 levels fell below 250 pmol/L.

Few women in the present study had a serum folate level indicative of folate deficiency. However, a sub-optimal vitamin B12 status may elevate serum concentration of folate. In order for folate to be retained by cells, folate must be converted from its monoglutamate form to its polyglutamate form. The preferred substrate for the enzyme that catalyzes this reaction is the tetrahydrofolate form of folic acid (22). Therefore, in vitamin B12 deficiency, if methyltetrahydrofolate cannot be converted to tetrahydrofolate, folate will not be retained by the cell. This condition results in high serum concentrations of folate in the face of low tissue folate levels.

There were several limitations in our study. The first was that the folate status was determined using serum samples as opposed to determining the red blood cell concentration of folate which would have provided a better indication of tissue folate status. Other nutrients, such as vitamin A and vitamin B6 that are associated with anaemia, were not measured. Subjects were also not tested for hookworm or other parasitic diseases besides malaria—a potential cause of anaemia due to intestinal blood loss. Although a comprehensive assessment of parasitic infections was not conducted in the study, such infections are common in pregnant women in Nigeria. Egwunyenga and co-workers (23) studied more than 2,000 pregnant women in Plateau State in north-central Nigeria and found that approximately 40% and 48% of the women were infected with malaria parasites and intestinal helminths respectively.

We conclude that a high percentage of the pregnant women we studied in Nigeria were anaemic. The main contributing factor to anaemia in these women was iron deficiency. Secondly, suboptimal vitamin B12 status may contribute to elevated concentrations of homocysteine, a risk factor for neural tube defects and pre-eclampsia. Once identified, either or both of these nutritional deficiencies could be corrected by food assistance programmes or supplementation of vulnerable groups in a population. An effective method for ensuring widespread correction of specific nutritional deficiencies could be accomplished by fortification of foods similar to the fortification with folate that is common in developed countries for the prevention of neural tube defects. Because of the importance of iron stores in maintaining iron balance during pregnancy, efforts should also be directed to improving the iron stores of young women prior to pregnancy.

## ACKNOWLEDGEMENTS

This work was supported by a Minority International Training Program grant funded by Fogarty International Center of the National Institutes of Health, USA.

## References

[1] World Health Organization (1992). The prevalence of anaemia in women: a tabulation of available information.

[2] Brabin BJ, Hakimi M, Pelletier D (2001). An analysis of anemia and pregnancy-related maternal mortality. J Nutr.

[3] van den Broek N (1998). Anaemia in pregnancy in developing countries. Br J Obstet Gynaecol.

[4] World Health Organization (1994). Prevention and management of severe anaemia in pregnancy: report of a technical working group.

[5] World Bank (1993). World development report 1993: investing in health.

[6] Ujah IAO, Uguru UE, Aisien AO, Sagay AS, Otubu JAM (1999). How safe is motherhood in Nigeria?: trends of maternal mortality in a tertiary health institution. East Afr Med J.

[7] Onwuhafua PI, Onwuhafua A, Adze J (2000). The challenge of reducing maternal mortality in Nigeria. Int J Gynaecol Obstet.

[8] Adamu YM, Salihu HM, Sathiakumar N, Alexander GR (2003). Maternal mortality in Northern Nigeria: a population-based study. Eur J Obstet Gynecol Reprod Biol.

[9] Baker SJ, DeMaeyer EM (1979). Nutritional anemia: its understanding and control with special reference to the work of the World Health Organization. Am J Clin Nutr.

[10] DeMaeyer E, Adiels-Tegman M (1985). The prevalence of anaemia in the world. World Health Stat Q.

[11] Fleming AF (1968). A study of anaemia in pregnancy in Ibadan, Western Nigeria, with special reference to folic acid deficiency.

[12] VanderJagt DJ, Spelman K, Ambe J, Datta P, Blackwell W, Crossey M (2000). Folate and vitamin B12 status of adolescent girls in northern Nigeria. J Natl Med Assoc.

[13] Burtis CA, Ashwood ER (2001). Tietz Fundamentals of clinical chemistry, 5th ed..

[14] van den Broek NR, Letsky EA, White SA, Shenkin A (1998). Iron status in pregnant women: which measurements are valid?. Br J Haematol.

[15] van den Broek NR, Letsky EA (2000). Etiology of anemia in pregnancy in south Malawi. Am J Clin Nutr.

[16] Lindenbaum J, Rosenberg IH, Wilson PW, Stabler SP, Allen RH (1994). Prevalence of cobalamin deficiency in the Framingham elderly population. Am J Clin Nutr.

[17] Vollset SE, Refsum H, Irgens LM, Emblem BM, Tverdal A, Gjessing HK (2000). Plasma total homocysteine, pregnancy complications, and adverse pregnancy outcomes: the Hordaland Homocysteine study. Am J Clin Nutr.

[18] Refsum H (2001). Folate, vitamin B12 and homocysteine in relation to birth defects and pregnancy outcome. Br J Nutr.

[19] Glew RH, Williams M, Conn CA, Cadena SM, Crossey M, Okolo SN (2001). Cardiovascular disease risk factors and diet of Fulani pastoralists of northern Nigeria. Am J Clin Nutr.

[20] Glew RH, Conn CA, VanderJagt TA, Calvin CD, Obadofin MO, Crossey MJ (2004). Risk factors for cardiovascular disease and diet of urban and rural dwellers in northern Nigeria. J Health Popul Nutr.

[21] VanderJagt DJ, Patel RJ, El-Nafaty AU, Melah GS, Crossey MJ, Glew RH (2004). High-density lipoprotein and homocysteine levels correlate inversely in preeclamptic women in northern Nigeria. Acta Obstet Gynecol Scand.

[22] Chanarin I, Deacon R, Perry J, Lumb M (1981). How vitamin B12 acts. Br J Haematol.

[23] Egwunyenga AO, Ajayi JA, Nmorsi OP, Duhlinska-Popova DD (2001). Plasmodium/intestinal helminth co-infections among pregnant Nigerian women. Mem Inst Oswaldo Cruz.

